# Slow Magnetic Relaxation in Silver(II) Macrocyclic Systems

**DOI:** 10.1021/acs.inorgchem.3c02265

**Published:** 2023-11-09

**Authors:** Joan Serra, Mercè Font-Bardia, Albert Escuer, Júlia Mayans

**Affiliations:** † Departament de Química Inorgànica i Orgànica, secció Inorgànica and Instutut de Nanociència i Nanotecnologia, Universitat de Barcelona, Martí i Franqués 1-11, Barcelona 08028, Spain; ‡ Departament de Mineralogia, Cristal·lografia i Dipòsits Minerals, Universitat de Barcelona, Martí Franqués s/n, Barcelona 08028, Spain; § Unitat de Difracció de R-X, Centre Científic i Tecnològic de la Universitat de Barcelona, Solé i Sabarís 1-3, Barcelona 08028, Spain

## Abstract

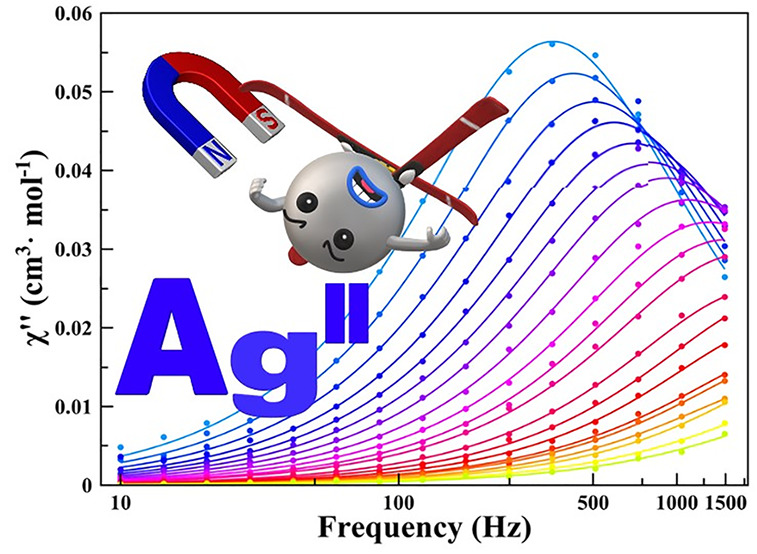

The spin–lattice relaxation time has been studied trough alternating-current susceptometry and ultralow-frequency Raman spectroscopy in a family of silver(II)-derived molecular systems with spin ^1^/_2_ and formulas [Ag^II^(*m*-CTH)(NO_3_)_2_] (**1**) and [Ag^II^(*m*-CTH)(ClO_4_)_2_] (**2**), where CTH = *meso*-5,5,7,12,12,14-hexamethyl-1,4,8,11-tetraazacyclotetradecane. The combination of both techniques demonstrates the occurrence of slow spin magnetic relaxation induced by spin–phonon interaction. The magnetic behavior of these silver(II)-derived systems opens the door to a new cation in the scarce family of *S* = ^1^/_2_ systems with slow relaxation of magnetization.

## Abstract 2

Silver(II) macrocyclic mononuclear compounds demonstrate field-induced slow relaxation of magnetization, expanding this magnetic response to a new element. Determination of the relaxation mechanisms through alternating-current susceptomery and terahertz Raman spectroscopy shows that relaxation goes through Raman plus a direct mechanism.

During the past few decades, the study of slow magnetic relaxation in paramagnetic molecular systems has been a hot topic in chemistry, physics, and nanotechnology.^1^ The first approaches to these kinds of molecules have recently been derived in proposing them as building blocks for a wide range of applications like spintronics,^2,3^ magnetic memories,^1^ or quantum information technologies.^4−6^ However, despite all of these ambitions in applying them in real technologies, the fundamentals of their behavior are still not completely understood in some aspects, and the field of molecular magnetism continues to surprise the magnetochemistry community with new achievements^7,8^ and fundamental findings toward the possibility of fully understanding the phenomena.^9,10^

Magnetic bistability resulting from large magnetic anisotropy and spin has been extensively studied in the so-called single-molecule magnets (SMMs), but other systems exhibiting slow relaxation of magnetization with different origins, usually under an external applied field, are of current interest. These kinds of molecules are (i) the so-called quasi-isotropic slow-relaxing molecules, mainly related with half-filled shell d^5^ (Mn^II^) or f^7^ (Gd^III^) cations,^11−15^ and (ii) the systems with *S* = ^1^/_2_, restricted to some VO^2+^, Cu^II^ derivatives, and one example for Ir^IV^ or Fe^III^.^16−21^ These families of molecules have in common that their slow magnetic relaxation cannot be explained as an overbarrier relaxation: the former because there is no possibility of significant anisotropy-dependent barrier and the latter because there are no available magnetic states apart from ±*m*_*S*_ = ^1^/_2_. In both cases, slow magnetic relaxation should be attributed to other magnetic relaxation paths. Among these *S* = ^1^/_2_ systems, vanadyl mononuclear compounds have been the more studied by far.^17−19^ A key parameter in slow-relaxing magnetic molecules is the so-called spin–lattice relaxation time (*T*_1_), which defines the time for the excited spin to relax to its ground state, and its control is required for further applications of magnetic molecules. It has been recently demonstrated^22−24^ that structural differences in closely related compounds can be of paramount importance in the magnetic relaxation properties of *S* = ^1^/_2_ systems and small differences in the structure can make less effective spin–lattice relaxation. Actually, it was suggested by Sessoli and co-workers that the magnetic field dependence of spin–lattice relaxation time in a family of V^IV^ mononuclear systems is also affected by a relaxation mechanism that involves low-energy vibrational modes (highly influenced by the mentioned structural differences, being especially relevant the rigidity of the system).^18^

Aliphatic macrocycles such cyclam, *m*-CTH, or *rac*-CTH (CTH = meso or racemic isomers of 5,5,7,12,12,14-hexamethyl-1,4,8,11-tetraazacyclotetradecane) are able to stabilize divalent silver complexes in which, with the exception of [Ag(*rac*-CTH)](ClO_4_)_2_, the anions usually link the elongated axial coordination sites of the cation (Figure S1).^25^ Being especially interested in *S* = ^1^/_2_ systems, we synthesized the complexes with formulas [Ag^II^(*m*-CTH)(NO_3_)_2_] (**1**) and [Ag^II^(*m*-CTH)(ClO_4_)_2_] (**2**) [see the Supporting Information (SI) for synthetic details], which exhibit higher stability than the *rac*-CTH or cyclam derivatives.

The dynamic properties for the Ag^II^ cation have only been studied for one silver(II) tetratolylporphyrin complex by pulsed electron paramagnetic resonance (EPR) in 1996,^26^ but the alternating-current (ac) response for this cation has never been measured maybe because Ag^II^ has been considered the weird analogue of the most studied Cu^II^ cation. However, it has large differences with respect to other *S* = ^1^/_2_ cations because it has electronic and nuclear spins of ^1^/_2_, moderate anisotropy, strong spin polarization of the neighboring ligands, and large spin delocalization, derived from its diffuse 4d orbitals, as has been shown by continuous-wave EPR experiments.^27−30^

In this Communication, we perform a systematic study of the mononuclear Ag^II^ systems **1** and **2** by means of ac magnetic susceptometry and ultralow-frequency Raman spectroscopy to study their slow magnetic relaxation and its relationship with low-energy vibrational modes. The study revealed differences in the spin dynamics of both compounds and proved that, for the very first time, the Ag^II^ cation could also present slow relaxation of magnetization.

Complexes **1** and **2** are mononuclear compounds (Figure 1) with the Ag^II^ cation in an axially elongated octahedral environment as a result of the expected Jahn–Teller distortion for a d^9^ configuration: four N atoms coming from the *m*-CTH ligand are coordinated in the short equatorial positions and to two weakly interacting O donors from the nitrate (**1**) or perchlorate (**2**) anions linked in the axial sites. Relevant structural information is summarized in Tables S1–S3 and Figures S2–S4.

**Figure 1:**
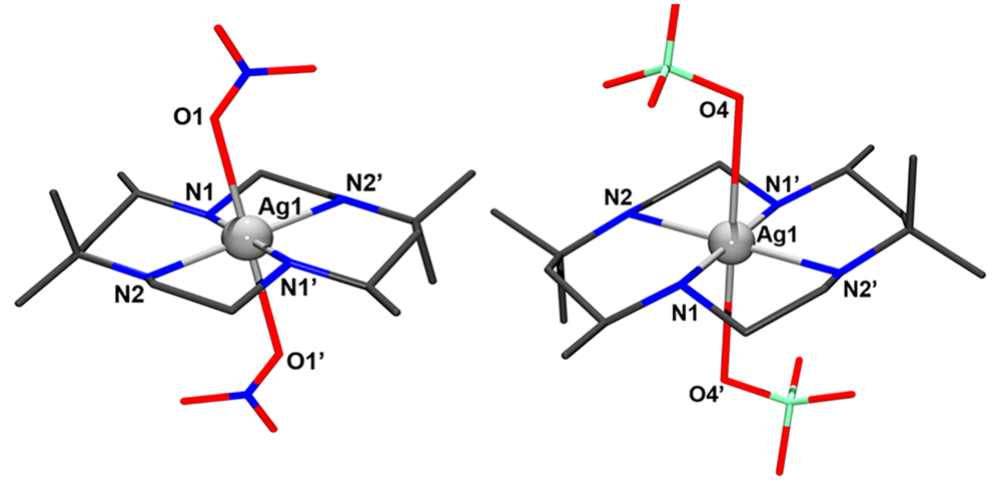
(Left) Labeled structure of **1**. (Right) Labeled structure of **2**. Color code: gray, Ag; navy, N; red, O; black, C; cyan, Cl. H atoms have been removed for clarity.

The representative parameter of the spin–lattice relaxation, τ (relaxation time), has been studied using ac susceptometry under different static magnetic fields for **1** and **2**. There is no imaginary component of the susceptibility (χ″) at zero field for either **1** or **2**, as is expected for *S* = ^1^/_2_. However, the application of a weak field allows slow relaxation of magnetization with the appearance of well-defined χ″(*T*) peaks (Figure S5). For **1**, the optimal field of 7000 G was selected to investigate the temperature dependence of τ, while 5000 G was the field chosen for **2** (Figure S6a,b for **1** and Figure 2a,b for **2**).

**Figure 2:**
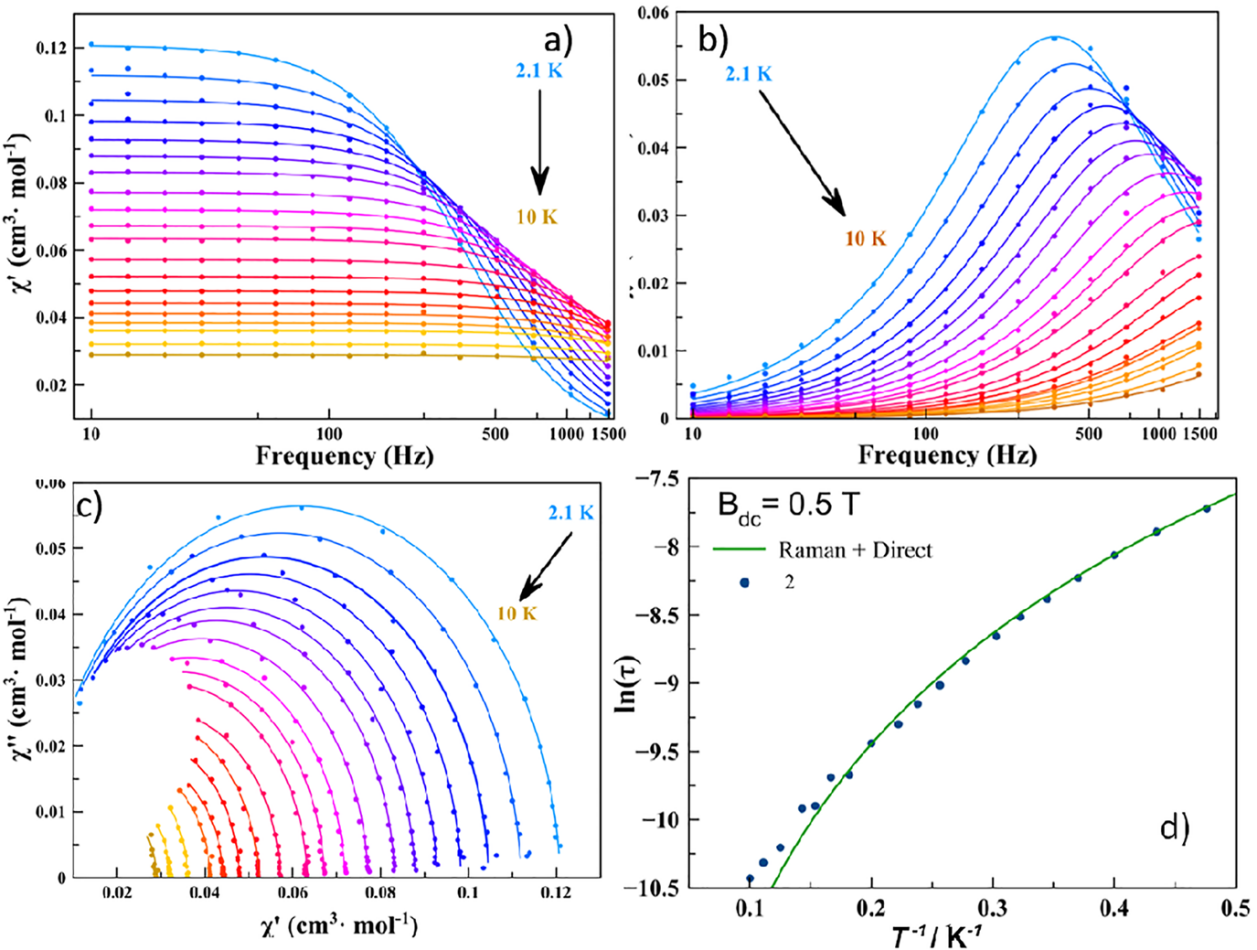
(a and b) Real and imaginary components of the magnetic susceptibility for **2** under an applied field of 5000 G. (c) Representation of the Argand plot. (d) Fitting of the Argand data using the Debye model.

The experimental data χ′(ν) and χ″(ν) of **1** and **2** were represented in the Argand plots (Figures S6c for **1** and Figure 2c for **2**) and fitted using the Debye model^30^ to extract the relaxation time dependence with temperature (Figure S6d for **1** and Figure 2d for **2**). It is clear from the logarithmic representation of τ versus *T* (Figure S7) that there is a very small change in the slope of the curves (Table S4). Indeed, it indicates a change in the nature of the main relaxation path. Because of the intrinsic nature of **1** and **2** (*S* = ^1^/_2_), the relaxation could not follow an Arrhenius law (see above) and the data have been fitted using the equation
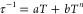
1with two contributions to the relaxation: the first term corresponds to the direct mechanism, which dominates at low temperature, and the second term is the Raman multiphonon process through virtual states, the thermally activated process.^31^ This model reproduces correctly the experimental behavior, and the best-fitting parameters are reported in Table S5. Interestingly, the value of the Raman exponents is *n* < 3 for **1** and **2**, much smaller than the predicted values for Kramers ions. However, similar Raman exponents have been reported for other *S* = ^1^/_2_ systems recently,^17−19^ indicating the intervention of acoustic (lattice) and optical (molecular) phonons.^17^

A deeper study on the magnetic relaxation of **2** has been done because of the clearer magnetic behavior probably due to significant structural and packing differences (see the SI for details). This compound has been extensively studied in the temperature range 2.2–7.5 K under different strengths of the applied magnetic field to relate the different relaxation paths with temperature and with the applied magnetic field (from 0.15 T up to 3 T because under higher fields the ac response vanishes (Figures S8–S10). The experimental values of τ, represented as isothermal scatters, are depicted in Figure 3. For all of the temperatures, τ increases with increasing magnetic field, reaches a maximum around 1.4 T, and then starts to decrease. This behavior of the relaxation rate with the magnetic field has two main contributions: At low fields, the relaxation is promoted by spin–spin and hyperfine interactions (inter- and intramolecular interactions), which are suppressed with increasing field. Instead, at higher fields, the relaxation is dominated by the spin–phonon direct mechanism (see the first term of eq 2) because more phonons with energy equal to separation of the *m*_*S*_ states are present. So, the relaxation time of **2** shows its maximum at intermediate fields when the direct relaxation mechanism has not been activated but it has been starting to suppress spin–spin and spin–nuclei interactions. As is shown in Figures 3 and S11, this behavior is temperature-independent.^17−19^ Considering these two different contributions of the magnetic field to the relaxation rate, the magnetic field dependence of τ (Table S6) has been fitted using the expression
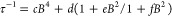
2where the first term is the field-dependent direct mechanism for *S* = ^1^/_2_ and the second term is related with the Brons–Van Vleck formula for Raman relaxation.^17,32,33^ The fitting curves at each temperature are depicted in Figure S12, and the best-fitting parameters are summarized in Table S7.

**Figure 3:**
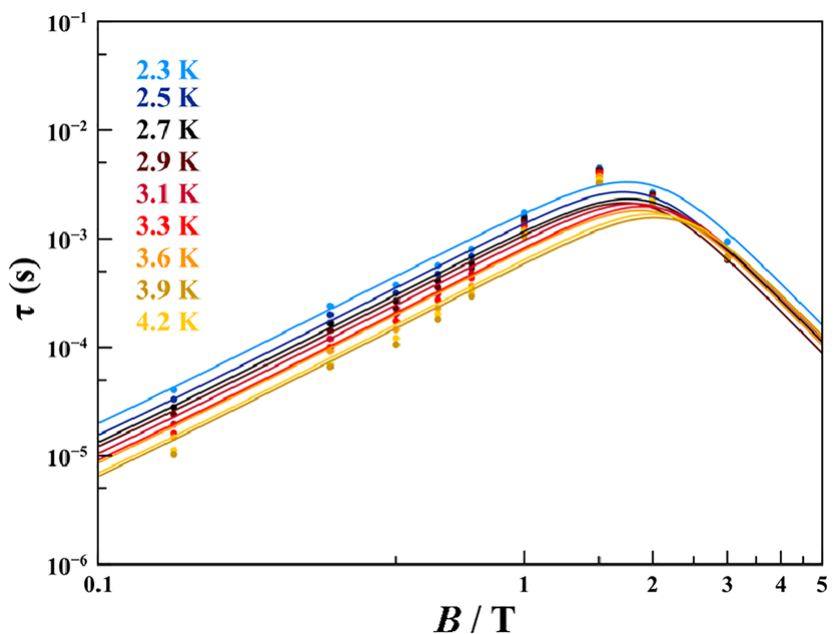
Dependence of τ with the magnetic field for **2** at different temperatures up to 4.2 K. The solid line represents the best fit.

The study of the dependence of τ with temperature under the application of different magnetic fields using the Debye model (Figures S9 and S10) reveals that, from 0.15 to 0.75 T, a combination of direct plus Raman mechanisms is needed to properly fit the experimental behavior, and even the direct mechanism should be more active at high fields. Among this, at very high fields (2 and 3 T), a contribution of quantum tunnelling of magnetization is needed, and even this mechanism is usually more active at low fields (Figure S10).

Even if the global relaxation could not follow in any case an Arrhenius relaxation, the *d* coefficient described in the second term of eq 2 is proportional to
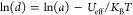
3and this is confirmed by the representation of ln(*d*) versus 1/*T* (Figure 4, inset), giving an energy effective barrier of *U*_eff_ = 9.10(8) cm^–1^ for the low-temperature range, which is compatible with the values of ultralow-frequency energy vibration modes (also the high-temperature range was fitted, but not reliable information was obtained; Figure S13). This hypothesis was validated in previous works by measuring IR spectroscopy in the terahertz frequency range and relating the molecular vibrations with spin–lattice relaxation with each vibrational mode coupled to the spin.^17−19,34^ Because, for **2**, vibrational modes should be involved in the magnetic relaxation path (see above), ultralow Raman spectroscopy (preferred to terahertz IR spectroscopy for centrosymmetric systems) was performed on **2** in the 1–30 cm^–1^ frequency region at room temperature, and several absorption peaks at 6.9, 13, and 17.5 cm^–1^ appeared (Figure 4). When the relationship *U*_eff_ = *h*ω/2 is applied, a reasonable correlation appears between the extracted activation energy of the *d* parameter and the energy of the Raman absorptions at similar energies, mainly the most intense peak at 6.9 cm^–1^.

**Figure 4:**
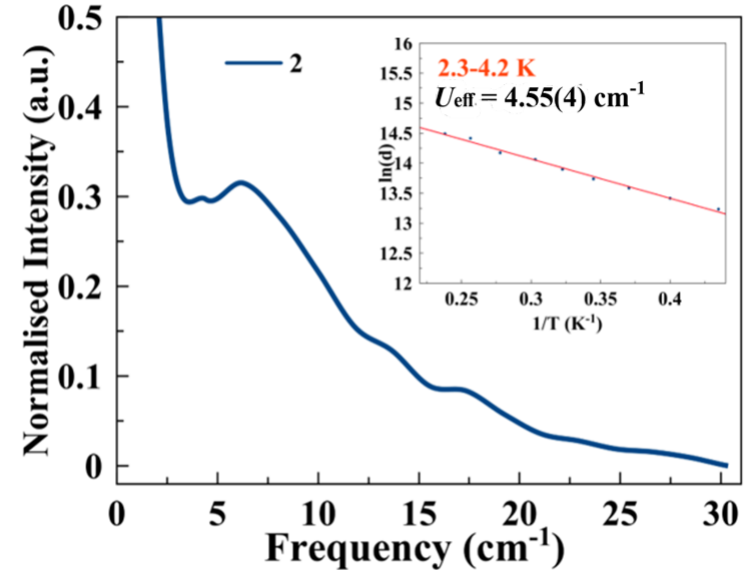
Raman spectra in the terahertz region of **2**. Inset: Arrhenius plot of the *d* parameter.

In conclusion, this study reveals that Ag^II^ is able to exhibit slow relaxation of magnetization and joins the selected *S* = ^1^/_2_ family of molecules that show this property. Due to the lack of a double-well barrier, no overbarrier relaxation can be involved and their relaxation should be described by different paths (Raman and direct). The dependence of the relaxation time on the strength of the applied direct-current magnetic field shows a contribution of the direct mechanism at any applied field and an important participation in the relaxation of low-frequency vibrational modes corroborated by ultralow-frequency Raman spectroscopy. The present work opens the door to use the polarizing cation Ag^II^, with the unusual *S* = ^1^/_2_ and *I* = ^1^/_2_ combination of electronic and nuclear spins, as a building block in different nanotechnological applications, mainly related with quantum technologies.

## Supporting Information Available

Experimental procedures, structural information, complete ac measurements, and fit details (PDF)
