# Supplementary material for: Slow Magnetic Relaxation in Silver(II) Macrocyclic Systems
Source: Inorg Chem. 2023 Nov 9;62(46):18804–8. doi: 10.1021/acs.inorgchem.3c02265 (PMC10664066; doi:10.1021/acs.inorgchem.3c02265)
Supplement: Supplementary file 1 — ic3c02265_si_001.pdf [file ic3c02265_si_001.pdf]

Supporting Information

" Slow magnetic relaxation in silver(II) macrocyclic systems."

Joan Serra,[§] Mercè Font-Bardia,[¥] Albert Escuer,[§] and Júlia Mayans*[§]

[§] Departament de Química Inorgànica i Orgànica, Secció Inorgànica and Instutut de Nanociència i Nanotecnologia (IN2UB). Universitat de Barcelona, Martí i Franquès 1-11, Barcelona-08028, Spain.

[¥] Departament de Mineralogia, Cristal·lografia i Dipòsits Minerals, Universitat de Barcelona, Martí Franqués s/n, 08028 Barcelona (Spain) and Unitat de Difracció de R-X. Centre Científic i Tecnològic de la Universitat de Barcelona (CCiTUB), Solé i Sabarís 1-3. 08028 Barcelona.

Corresponding Author E-mail: julia.mayans@ub.edu

Table of contents

1- Experimental.

1.1 Physical measurements
1.2 Synthesis

2- Structural information

Figure S1. *Meso*-CTH (right) and *rac*-CTH showing the different arrangement of the methyl groups and the H-atoms linked to the nitrogen donors.
Table S1. Crystal data of the X-ray structures for **1** and **2**.
Figure S2. Partially labelled molecular structure for complexes **1** and **2.**
Table S2. Selected bond lengths (Å) and angles (º) for complex **1**.
Table S3. Selected bond lengths (Å) and angles (º) for complex **2**.
Figure S3. Coordination environment for the Ag^{II} cation in complexes **1** and **2**.
Figure S4. View of the unit cell along [010] direction for complexes **1** and **2**.

3- Ac data

Figure S5. Temperature dependence of the imaginary component of the magnetic susceptibility for **1** and **2** as a function of the applied magnetic field at a fixed frequency of 1000 Hz.
Figure S6. Real and imaginary components of the magnetic susceptibility, representation of the Argand plot and fit of the Argand plot for **1**.
Figure S7. Logarithmic temperature dependence of the relaxation time for compounds **1** and **2.**
Table S4. Slopes in the low temperature region (2-4.2 K) and in the high temperature region (4.2-7 K) for complexes **1** and **2.**
Table S5. Best fit parameters for **1** (7000 G) and for **2** (5000 G) used to reproduce the relaxation rate dependence with temperature.
Figure S8. Frequency dependence of χ' and χ'' of compound **2** at 0.15, 0.35, 0.5, 0.625, 0.75, 1.0, 1.5, 2.0 and 3.0 T.
Figure S9. Cole-cole plots of compound **2** at 0.15, 0.35, 0.5, 0.625, 0.75, 1.0, 1.5, 2.0 and 3.0 T.
Figure S10. Fitted plots of the temperature dependence of the relaxation times for compound **2** as a function of the applied magnetic field.
Figure S11. Magnetic field dependence of τ extracted from *ac* susceptibility measurements for compound **2** for the extended range of temperatures up to 7.5 K.
Figure S12. Magnetic field dependence of τ^{-1} extracted from *ac* susceptibility measurements for compound **2** at different temperatures.
Figure S13. Arrhenius plot of *d* parameters extracted from the fit of the Brons - van Vleck model.
Table S6. Tau vs. temperature values for compound **2** at the measured fields.
Table S7. Best-fit parameters of the model used to reproduce the field dependence of the magnetization relaxation for **2**.

1- **Experimental**.

1.1 Physical measurements

All manipulations were performed under aerobic conditions. All reagents and solvents were used as received. The magnetic susceptibility measurements were performed with a Quantum Design MPMS-5 SQUID magnetometer at the CCiT-Magnetochemistry Unit of the University of Barcelona. The two complexes are indefinitely stable in the mother solution and kept in the freezer. Solid complex **2** is stable at room temperature whereas complex **1** partially degrades in few hours. Thus, the magnetic measurements were performed on a pressed powdered sample for **2** and on a sample of **1** still wet of the mother solution and placed in a sealed holder. Freezing of the solvent keeps immobilized the sample avoiding rotation induced by the external field. Infrared spectra (4000-400 cm–1) were recorded from KBr pellets on a Bruker IFS-125 FT-IR spectrophotometer. Ultra-low frequency Raman spectra were recorded with a high resolution Raman T64000 (Jobin Yvon) instrument.

Single crystals of compounds **1** and **2** were set up on a Bruker D8-VENTURE diffractometer equipped with a multilayer monochromator and a Mo microfocus (λ = 0.71073 Å). The frames were integrated with the Bruker SAINT software package using a narrow-frame algorithm and the structures were solved and refined using the Bruker SHELXTL Software Package.

1.2 Synthesis

Complex **1** [Ag^{II}(*m*-CTH)$(NO_3)_2$]. *Meso*-CTH (1.081g, 3.8 mmol) and $AgNO_3$ (1.308g, 7.7mmol) were dissolved in 20 ml of MeOH under stirring during 2h. The formation of metallic silver was observed immediately. After that time, the mixture was centrifuged before decanting a yellow solution. The metallic silver remaining on the bottom of the tube was washed 2 times by centrifugation with methanol, which was added to the first fraction decanted before. A yellow powder was obtained after the addition of 200ml of diethyl ether and keeping the solution in the freezer overnight. Single crystals of **1** suitable for single crystal X-ray diffraction analyses were obtained by layering a methanolic solution of **1** with diethyl ether. Calc/found CNH for $C_{16}H_{36}AgN_6O_6$: C, 37.22/37.1; N 16.28/16.4; H 7.03/7.0.

Caution! The employment of perchlorates with organic solvents is potentially dangerous and must be handled carefully. Complex **2** [Ag^{II}(*m*-CTH)$(ClO_4)_2$] was synthesized by a similar procedure to **1** using $AgClO_4$ instead of $AgNO_3$ (1.6 g $AgClO_4$, 7.7 mmol). An orange powder was obtained after the addition of 200 ml of diethyl ether and keeping the solution in the freezer overnight. Single crystals of **2** suitable for single crystal X-ray diffraction analyses were obtained by diffusion of diethyl ether into a small vial containing an acetonitrile solution of **2**. Calc/found CNH for $C_{16}H_{36}AgCl_2N_4O_8$: C, 32.50/32.3; N 9.48/9.5.4; H 6.14/6.2.

IR spectra: coordinated NH 3420; NH 3290, 3256, 3150; CH 2930, 2865; 1625, 1463,1431,1144, 994,940, 890, 870. In addition, for **1,** NO_3^- 1385; for **2**, CLO_4^- 1090, 626 cm^{-1}.

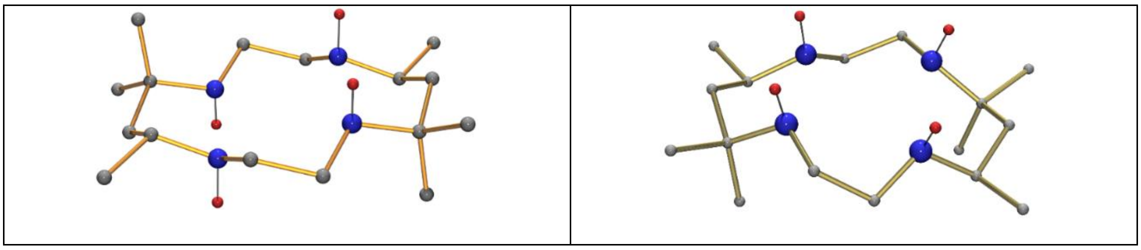

Figure 1. *Meso*-CTH (right) and *rac*-CTH showing the different arrangement of the methyl groups and the H-atoms linked to the nitrogen donors.

2- Structural information.

Table S1. Crystal data of the X-ray structures for **1** and **2**.

	**1**	**2**
**Formula**	$C_{16}H_{36}AgN_6O_6$	$C_{16}H_{36}AgCl_2N_4O_8$
**FW**	516.38	591.26
**System**	Monoclinic	Monoclinic
**Space group**	C 2/c	P 21/n
***a*/Å**	13.0051(9)	8.1987(6)
***b*/Å**	11.6620(8)	8.9468(6)
***c*/Å**	15.418 (1)	17.208(1)
***α*/deg.**	90	90
***β*/deg.**	112.477(3)	102.874(2)
***γ*/deg.**	90	90
**V/Å^3**	2160.7(3)	1230.5(2)
Z	4	2
***T*, K**	100(2)	100(2)
**λ(MoKα), Å**	0.71073	0.71073
**ρ_{calc}, g·cm^{-3}**	1.587	1.596
**μ(MoKα), mm^{-1}**	0.977	1.083
**F (000)**	1076	610
R	0.0752	0.0649
**ωR^2**	0.2375	0.2264

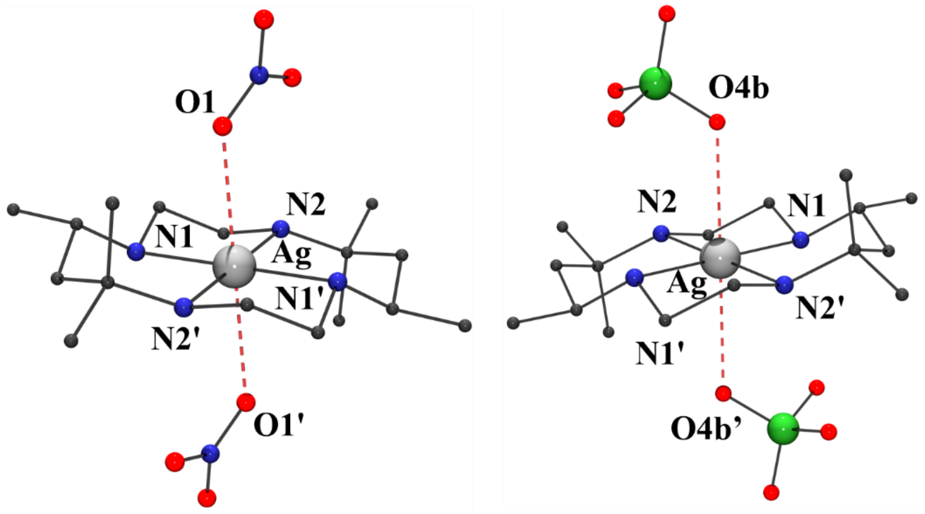

Figure S2. Partially labelled molecular structure for complexes **1** and **2**.

Table S2. Selected bond lengths (Å) and angles (º) for complex **1**. The structure of complex **1** was previously reported in 1978[1] (CCDC refcode TZTDAG) and solved in the same space group and giving similar cell parameters. However, the previously Ag-O (2.808 Å) and Ag-N bond distances (2.159/2.162 Å) were larger than those found in the current structural analysis.

**Ag1-N1**	2.116(3)	**O1-Ag-N1**	83.9(2)
**Ag1-N2**	2.108(3)	**O1-Ag-N2**	83.3(1)
**Ag1-O1**	2.678(6)	**O1-Ag-N1’**	96.1(2)
**N1-Ag-N2**	89.0(1)	**O1-Ag-N2’**	96.7(1)
**N1-Ag-N2’**	91.0(1)		

Table S3. Selected bond lengths (Å) and angles (º) for complex **2**. The perchlorate anion shows a small disorder and the reported Ag1-$O_{perchlorate}$ distance refers to the O4b position.

**Ag1-N1**	2.133(4)	**O4b-Ag-N1**	82.8(4)
**Ag1-N2**	2.142(4)	**O4b-Ag-N2**	77.9(4)
**Ag1-O4b**	2.721(18)	**O4b-Ag-N1’**	97.2(4)
**N1-Ag1-N2**	86.0(1)	**O4b-Ag-N2’**	102.1(4)
**N1-Ag1-N2’**	94.0(1)		

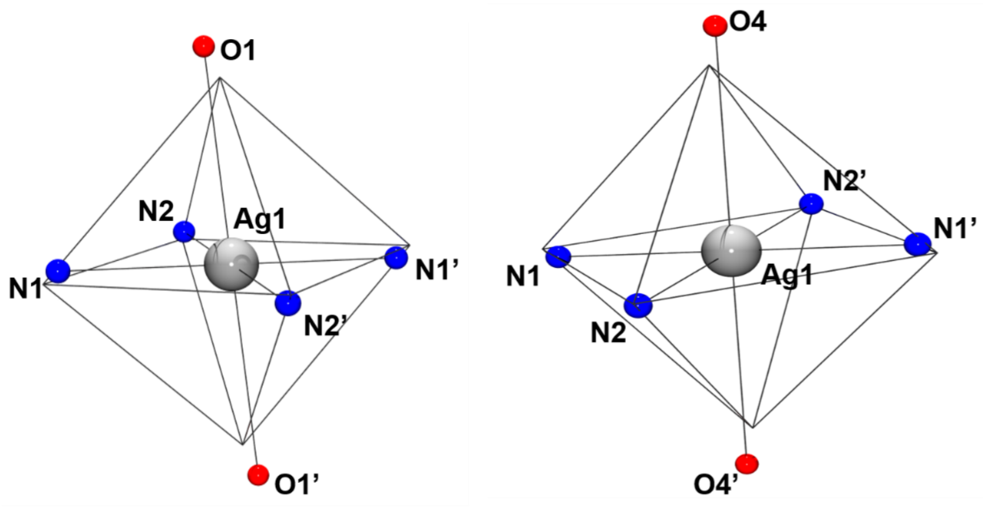

Figure S3. Coordination environment for the Ag^{II} cation in complexes **1** and **2**. SHAPE[2] analysis indicate a deviation coefficient from the regular octahedron of 1.75 and 2.12, respectively derived from the Jahn-Teller distortion and the slightly tilted position of the nitrato and perchlorato ligands due to the steric hindrance with the methyl groups. The grey lines correspond to the ideal octahedral geometry.

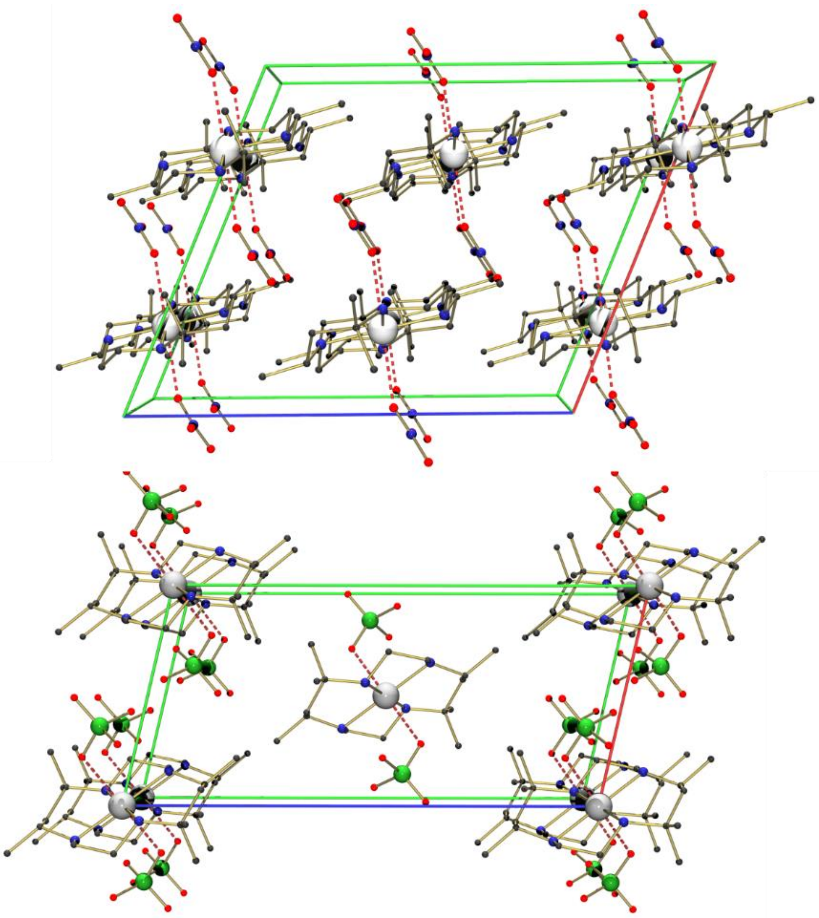

Figure S4. View of the unit cell along [010] direction for complexes **1** (top) and **2** (bottom). Color code: Siver, gray, N, navy; O, red; C, black; Cl, green; *z* axis, blue; *x* axis, red.

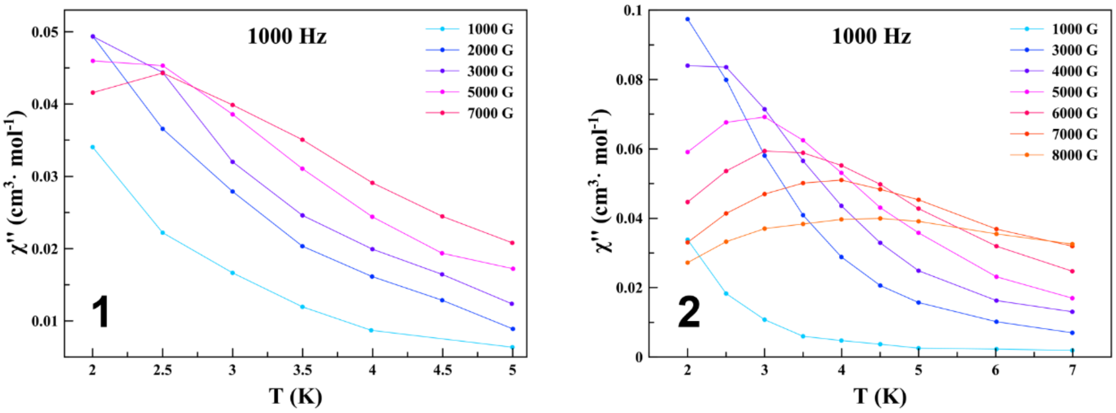

Figure S5. Temperature dependence of the imaginary component of the magnetic susceptibility for **1** (left) and **2** (right) as a function of the applied magnetic field field at a fixed frequency of 1000 Hz.

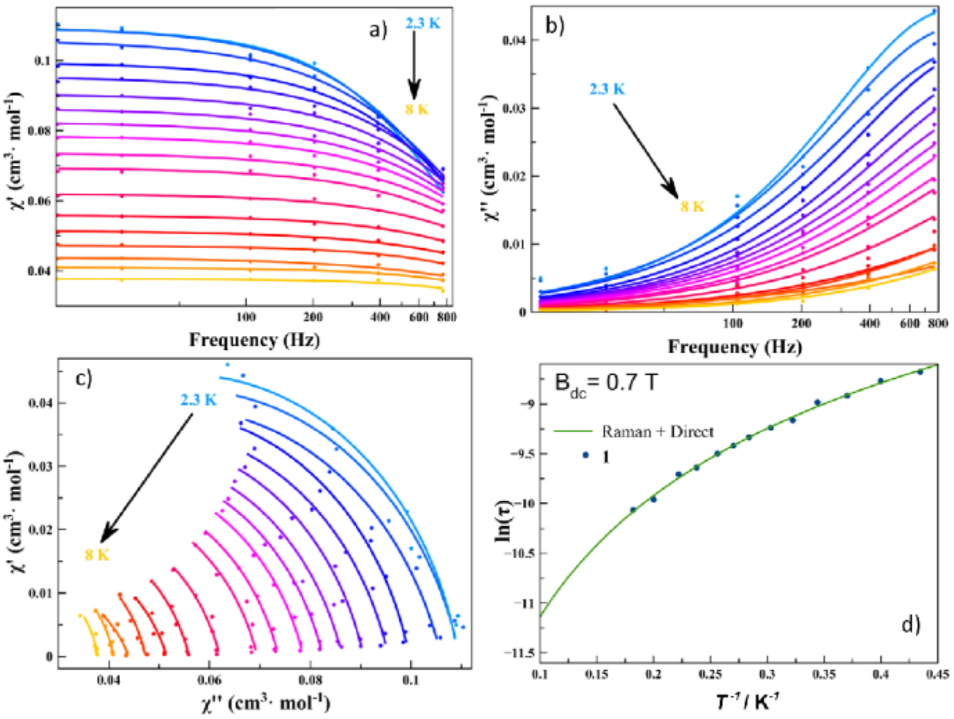

Figure S6. a) and b) real and imaginary components of the magnetic susceptibility for **1** under an applied field of 7000 G. c) representation of the Argand plot and d) fit of the Argand data using the Debye model. Data has been limited to 800 Hz due to the noisy plot at higher frequencies.

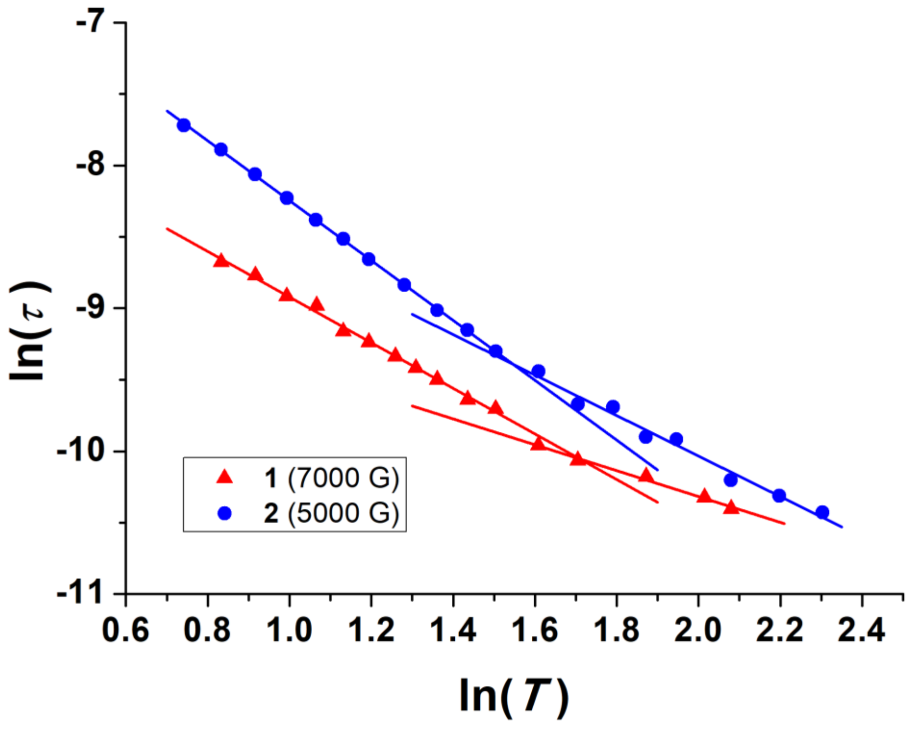

Figure S7. Logarithmic temperature dependence of the relaxation time for compounds **1** (red triangles) and **2** (blue circles) indicating a different dependence of the relaxation time at low and high temperatures. This dependence is a consequence of the different dominancy of the relaxation mechanisms involved in each temperature region.

Table S4. Slopes in the low temperature region (2.2-4.5 K) and in the high temperature region (4.5-10 K) for complexes **1** and **2.**

**Complex**	Low temperature region	High temperature region
**1**	-1.59 (*R* = 0.996)	-0.91 (R = 0.990)
**2**	-1.42 (R = 0.979)	-2.09 (R = 0.999)

Table S5. Best fit parameters for **1** (7000 G) and for **2** (5000 G) used to reproduce the relaxation rate dependence with temperature.

	**1**	**2**
a ($s^{-1}K^{-1}$)	1092.8(2)	153.8(3)
b ($s^{-1}K^{-n}$)	636.6(1)	463.4(1)
n	2.0	2.3

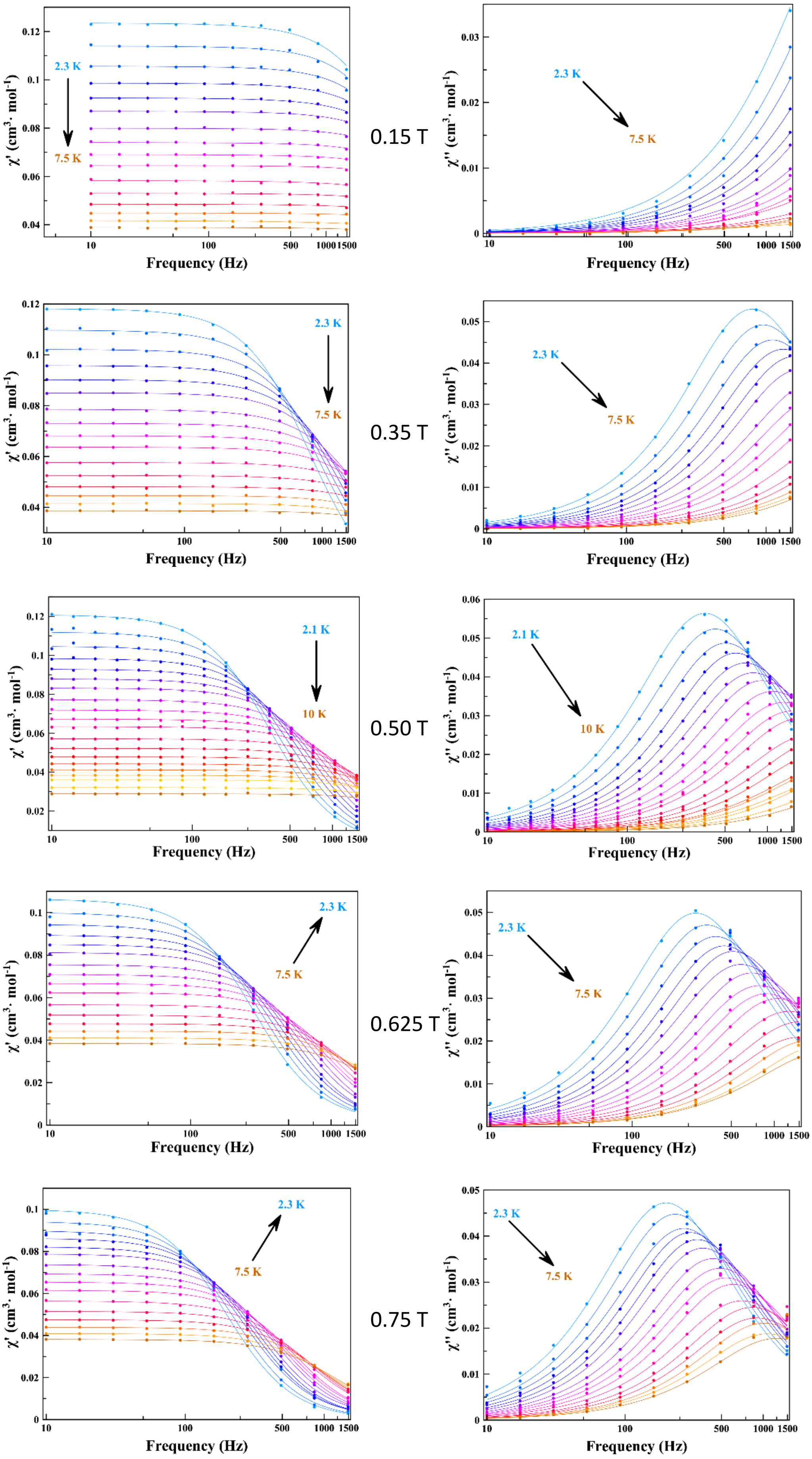

0.15 T
0.35 T
0.50 T
0.625 T
0.75 T
χ' (cm³· mol⁻¹)
χ'' (cm³· mol⁻¹)
Frequency (Hz)
2.3 K
7.5 K
2.1 K
10 K

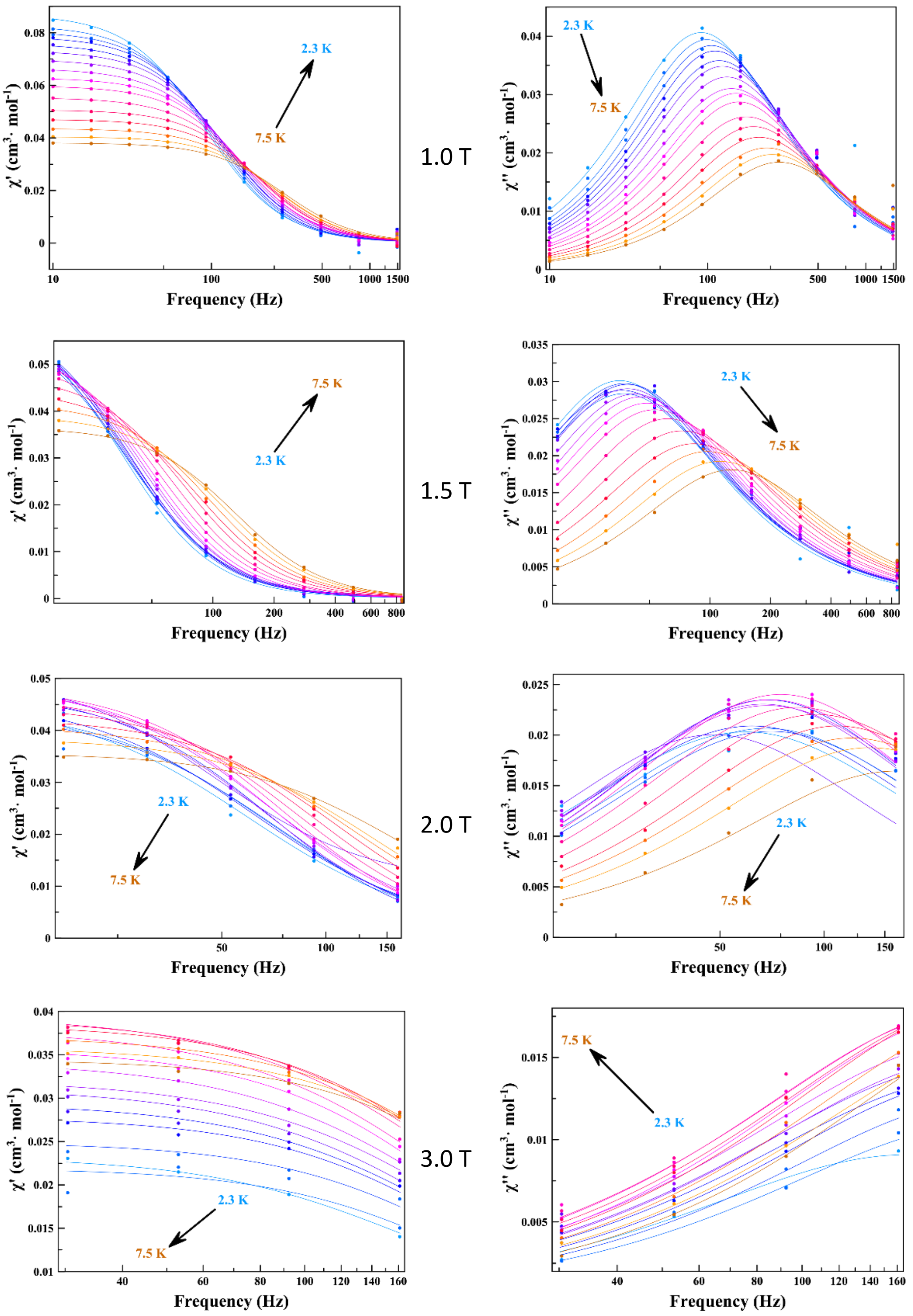

Figure S8. Frequency dependence of χ’ (left column) and χ’’ (right columns) of compound **2** at 0.15, 0.35, 0.5, 0.625, 0.75, 1.0, 1.5, 2.0 and 3.0 T. Solid lines show the best fit of the plots.

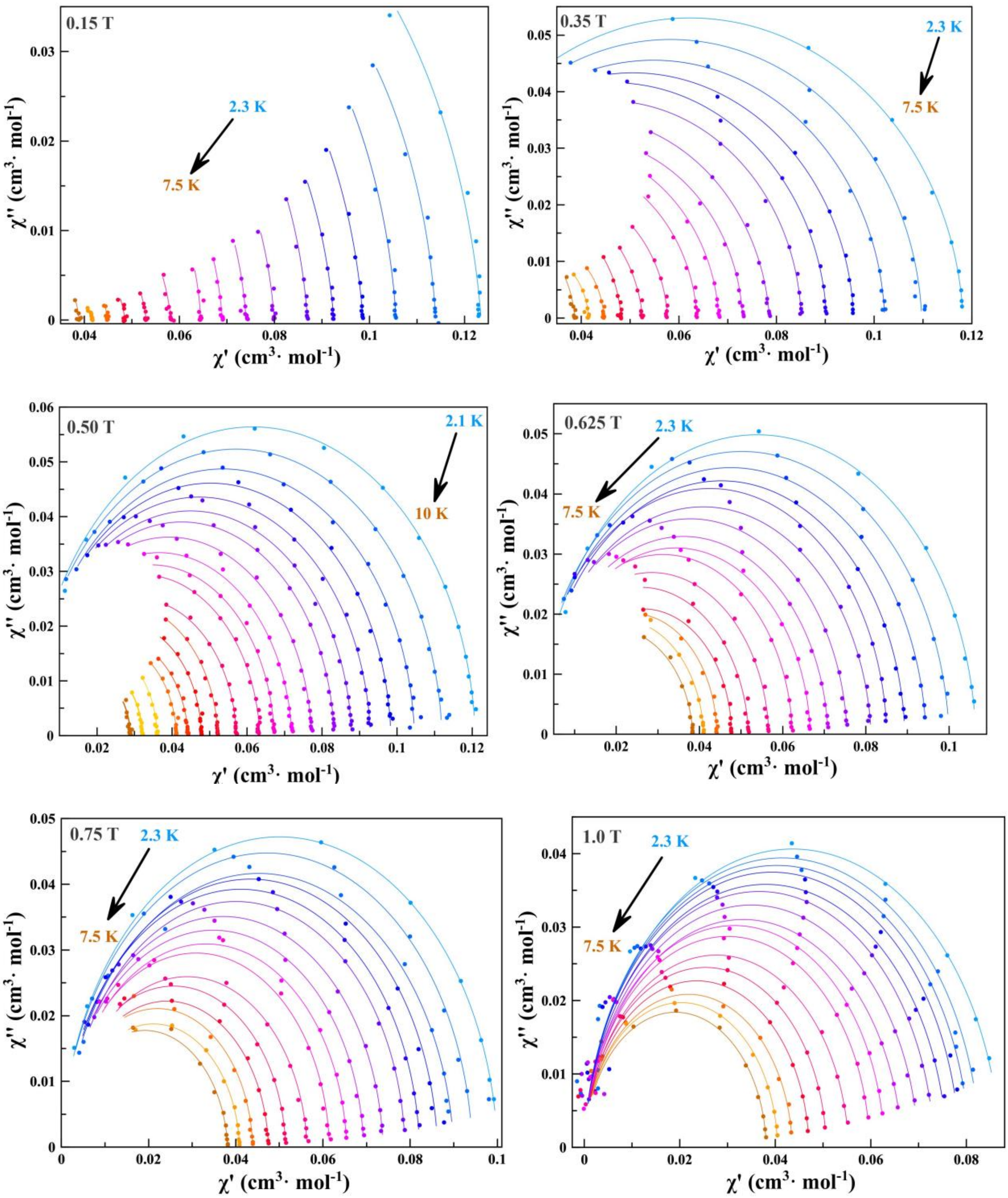

0.15 T
2.3 K
7.5 K
0.35 T
2.3 K
7.5 K
0.50 T
2.1 K
10 K
0.625 T
2.3 K
7.5 K
0.75 T
2.3 K
7.5 K
1.0 T
2.3 K
7.5 K
χ'' (cm³· mol⁻¹)
χ' (cm³· mol⁻¹)

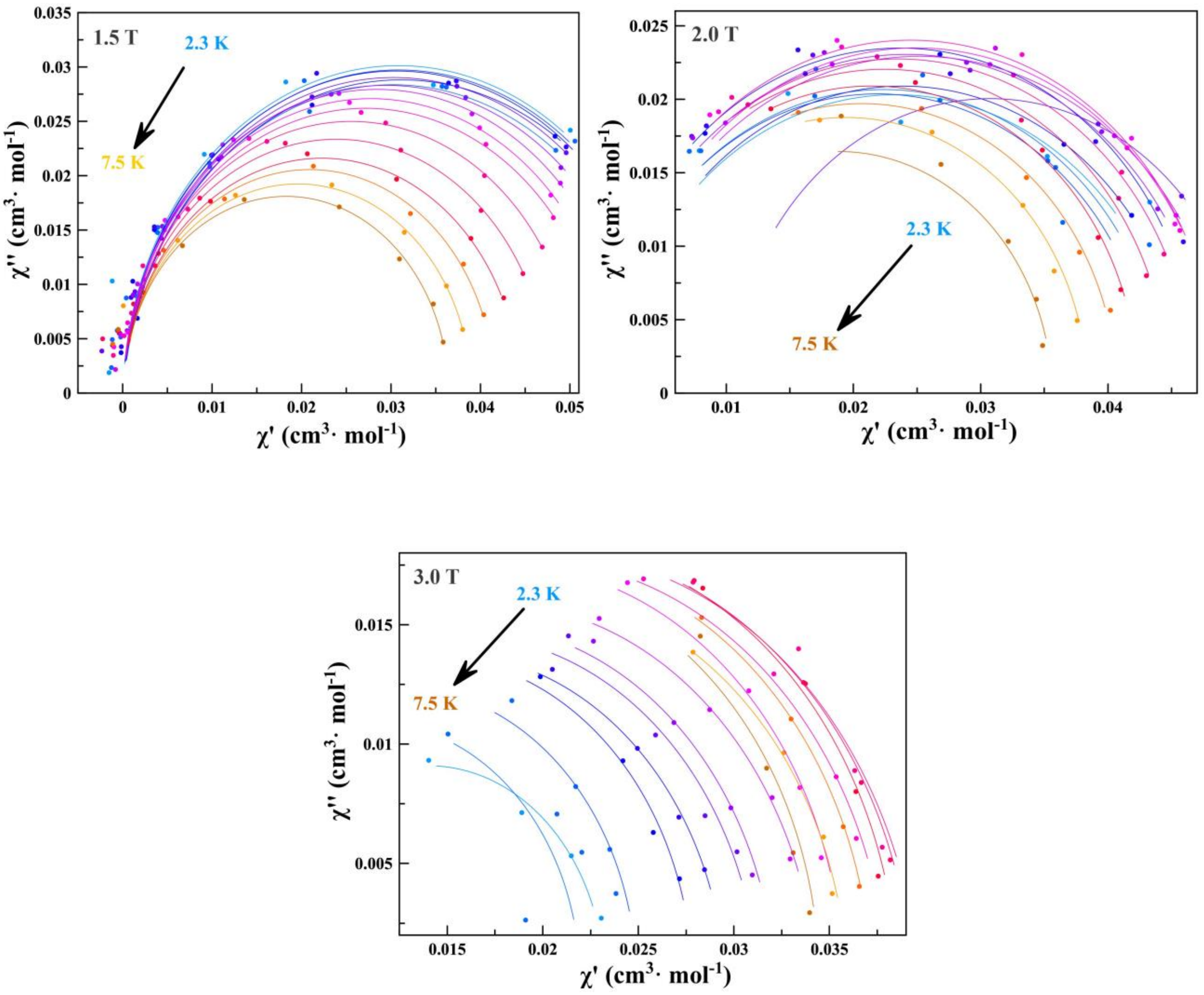

Figure S9. Cole-Cole plots of compound **2** at 0.15, 0.35, 0.5, 0.625, 0.75, 1.0, 1.5, 2.0 and 3.0 T. Solid lines show the best fit of the plots.

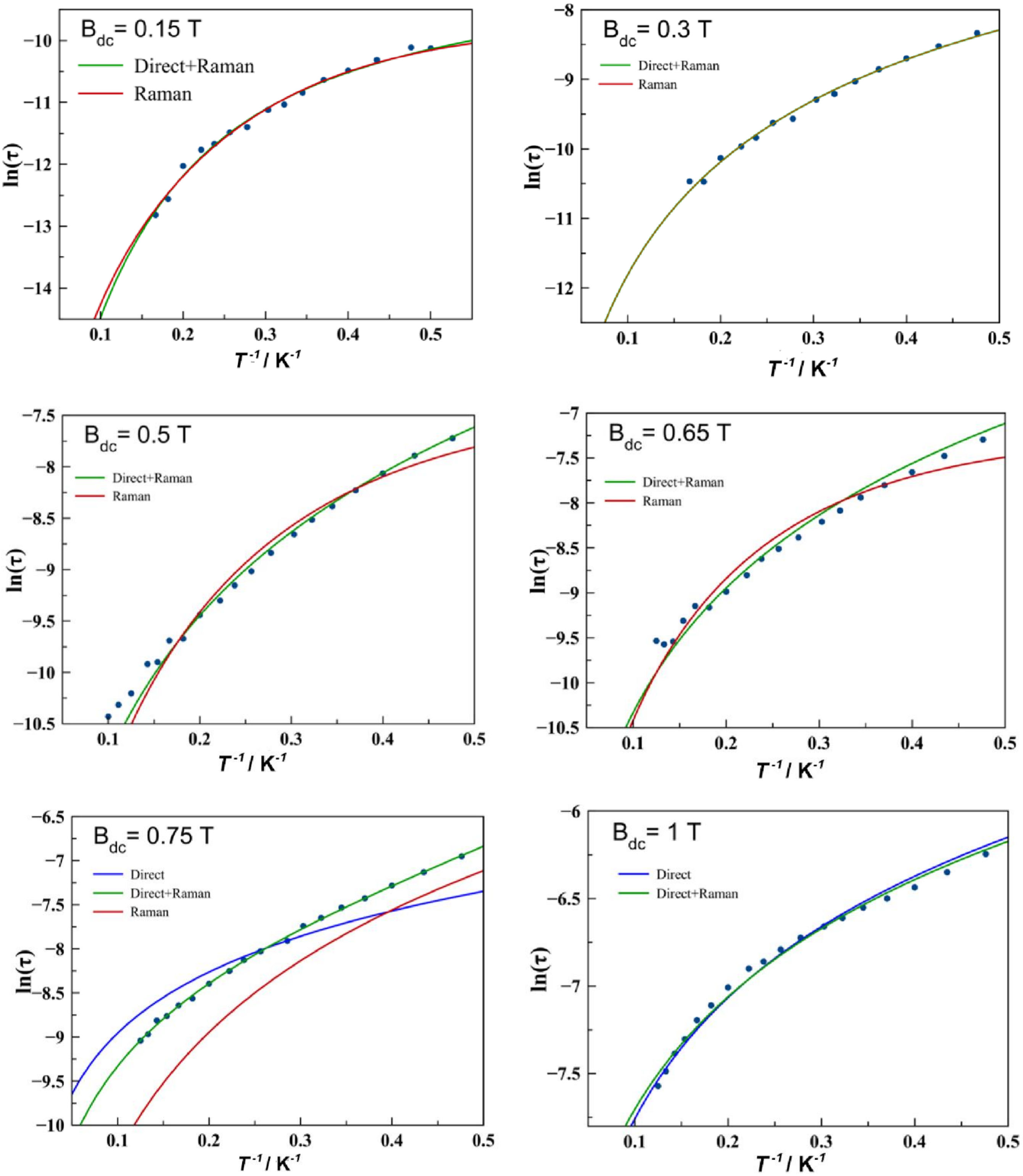

B_dc = 0.15 T
Direct+Raman
Raman
ln(τ)
T^-1 / K^-1
B_dc = 0.3 T
Direct+Raman
Raman
ln(τ)
T^-1 / K^-1
B_dc = 0.5 T
Direct+Raman
Raman
ln(τ)
T^-1 / K^-1
B_dc = 0.65 T
Direct+Raman
Raman
ln(τ)
T^-1 / K^-1
B_dc = 0.75 T
Direct
Direct+Raman
Raman
ln(τ)
T^-1 / K^-1
B_dc = 1 T
Direct
Direct+Raman
ln(τ)
T^-1 / K^-1

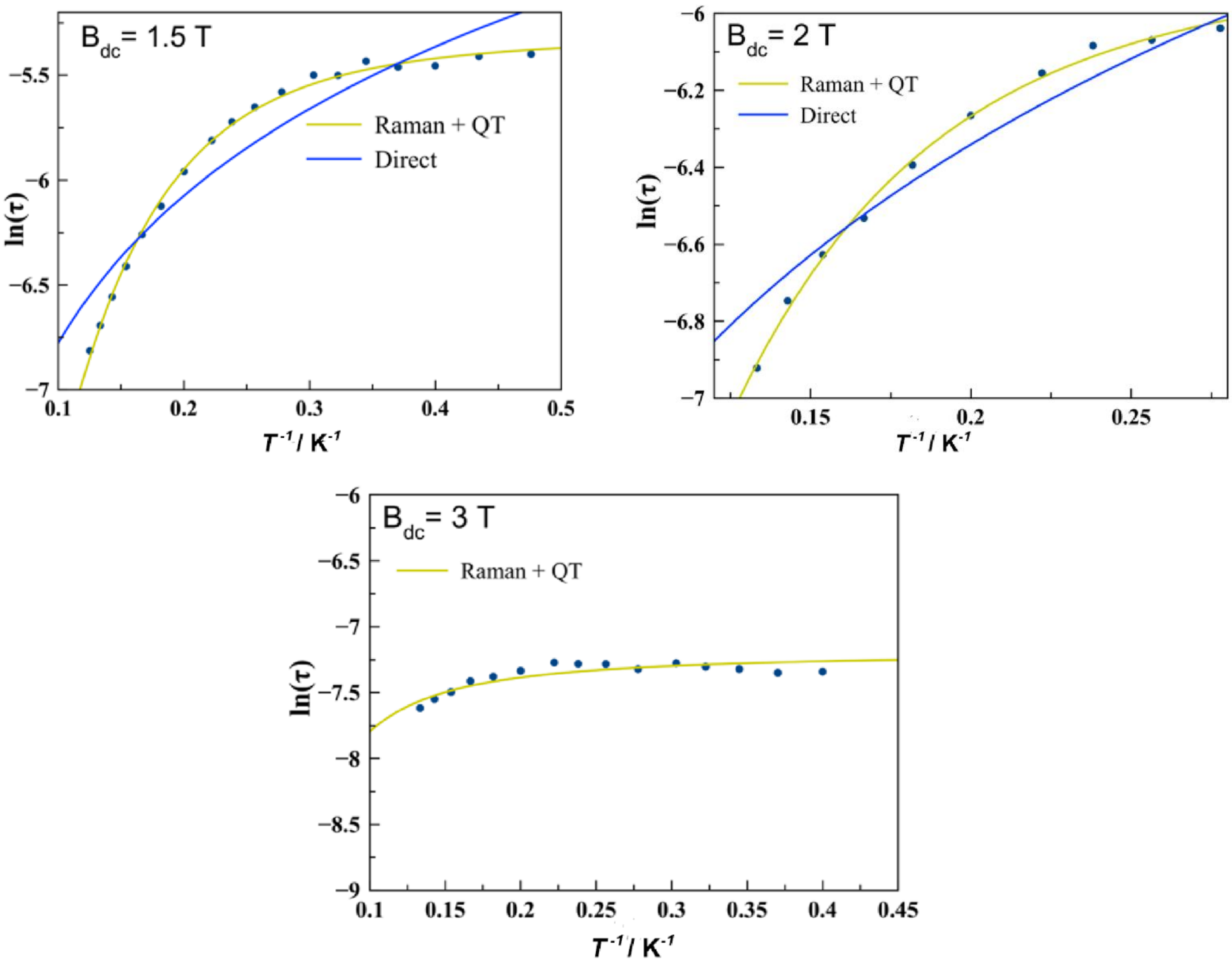

Figure S10. Fitted plots of the temperature dependence of the relaxation times for compound **2** as a function of the applied magnetic field. Solid lines show the best fit of the plots.

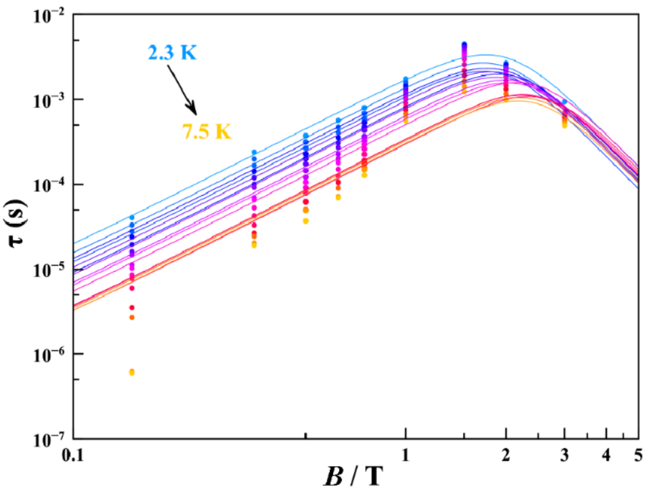

Figure S11. Magnetic field dependence of τ extracted from *ac* susceptibility measurements for compound **2** for the extended range of temperatures up to 7.5 K. Solid lines are the best-fits parameters.

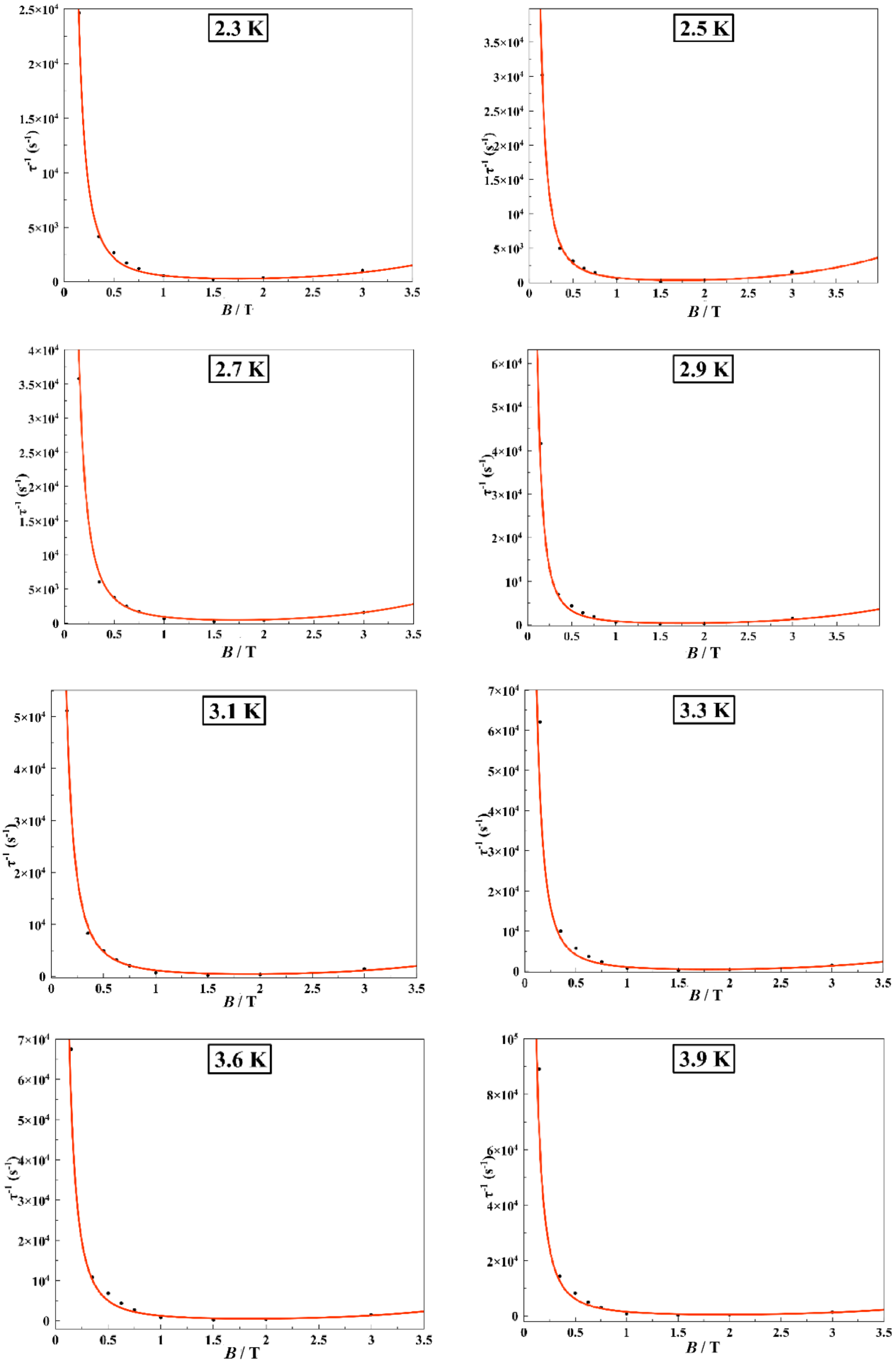
2.3 K
2.5 K
2.7 K
2.9 K
3.1 K
3.3 K
3.6 K
3.9 K
τ^{-1} (s^{-1})
B / T

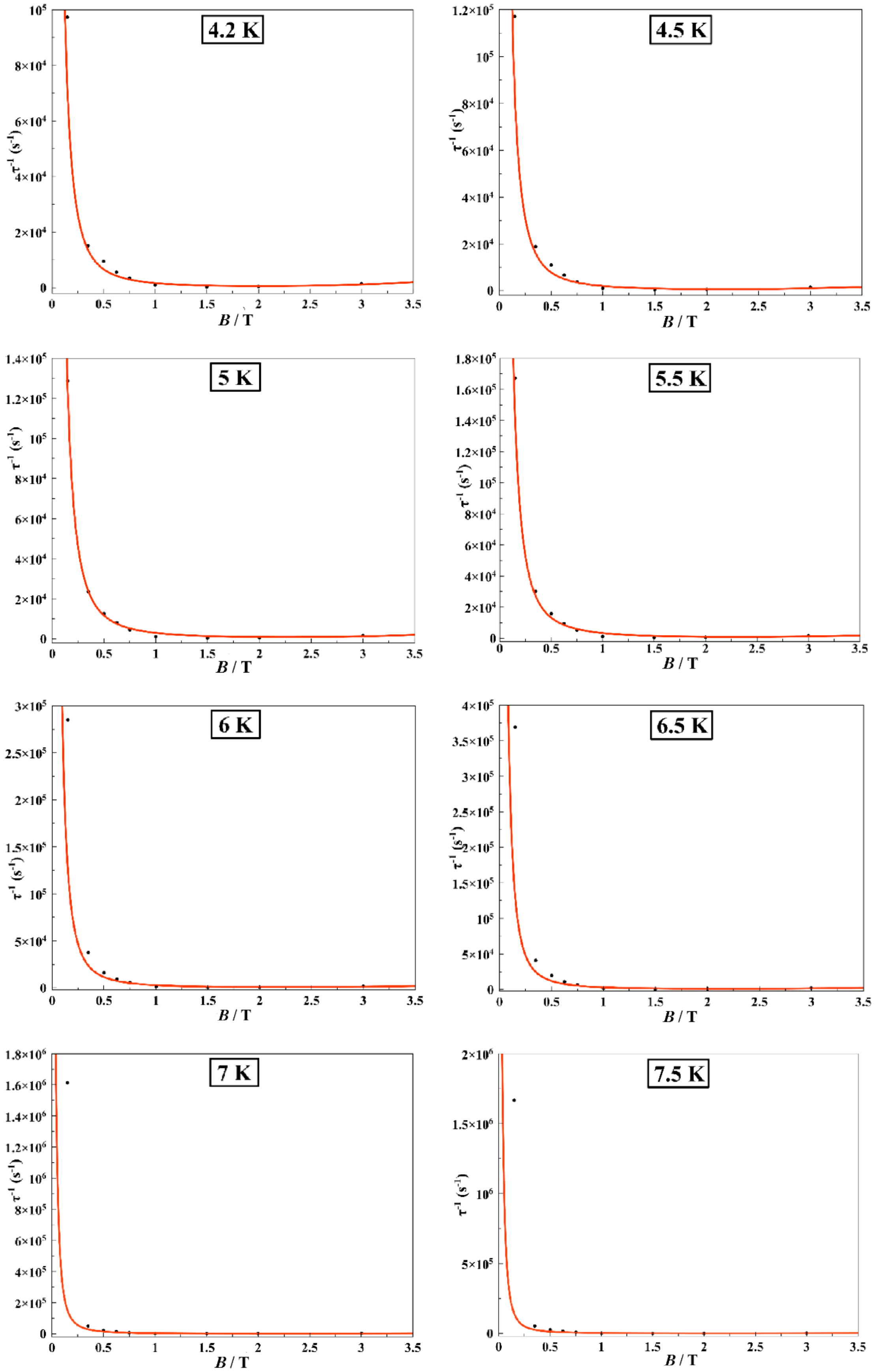

Figure S12. Magnetic field dependence of τ^{-1} extracted from *ac* susceptibility measurements for compound **2** at different temperatures. Red lines are the best-fits models.

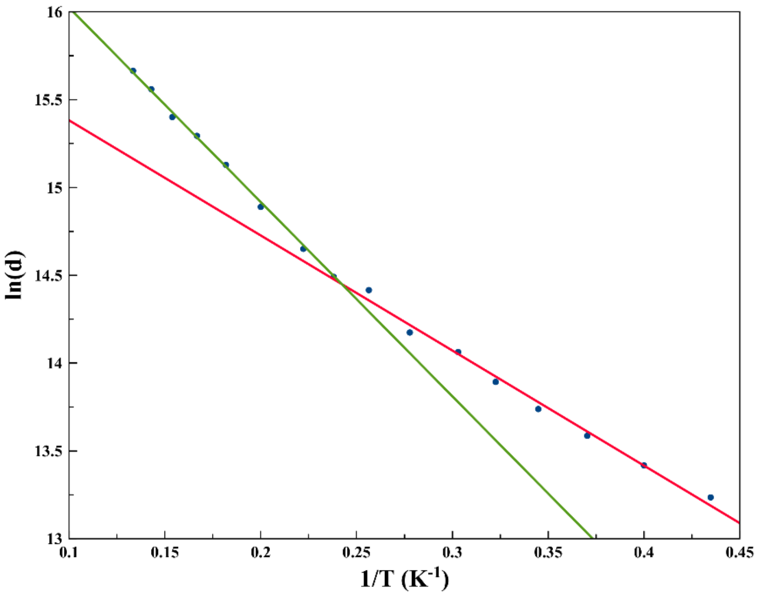

Figure S13. Arrhenius plot of *d* parameters extracted from the fit of the Brons - van Vleck model. Two different tendencies can be fitted depending on the temperature regions (red line between 2.3-4.2 K and green line between 4.5-7.5 K).

Table S6. Tau vs. temperature values for compound **2** at the measured fields.

**1500 G**		**3500 G**	
T	tao	T	tao
1.9992	4.00492E-5	2.1	2.402274985453999E-4
2.09924	4.05962E-5	2.3	1.989553870602539E-4
2.29976	3.31503E-5	2.5	1.668542089722929E-4
2.4997	2.79672E-5	2.7	1.426288076793638E-4
2.6998	2.40289E-5	2.9	1.198307333941803E-4
2.8997	1.95499E-5	3.1	1.0009744534477352E-4
3.0998	1.61206E-5	3.3	9.236392265664423E-5
3.30031	1.48112E-5	3.6	7.007822849362737E-5
3.60129	1.12155E-5	3.9	6.605217360889614E-5
3.89823	1.02786E-5	4.2	5.339438832857871E-5
4.20197	8.54419E-6	4.5	4.711866259415894E-5
4.50504	7.7668E-6	5.0	3.981511037748051E-5
4.99991	5.98302E-6	5.5	2.8314503597055093E-5
5.4996	3.505E-6	6.0	2.8464751659933698E-5
5.99961	2.7095E-6		

**5000 G**		**6250 G**	
T	tao	T	tao
2.1	4.434177903550548E-4	2.1	6.796790775326833E-4
2.3	3.743441209870697E-4	2.3	5.664733487825328E-4
2.5	3.149657947909435E-4	2.5	4.729234310325563E-4
2.7	2.6692944778161415E-4	2.7	4.089985406584857E-4
2.9	2.28725461658703E-4	2.9	3.566682157362913E-4
3.1	2.0056510855173123E-4	3.1	3.083570355562935E-4
3.3	1.738820959349717E-4	3.3	2.7241205660606975E-4
3.6	1.4530648857275543E-4	3.6	2.2872086455184786E-4
3.9	1.2149519746523347E-4	3.9	2.0141689065195187E-4
4.2	1.0590318937022292E-4	4.2	1.8009418840767195E-4
4.5	9.134216580736272E-5	4.5	1.5036140846905798E-4
5.0	7.94212289753307E-5	5.0	1.2517484925787987E-4
5.5	6.304152075840242E-5	5.5	1.0492617020825478E-4
6.0	6.187145306008342E-5	6.0	1.066542253484029E-4
6.5	5.020625868690145E-5	6.5	9.044682346290279E-5
7.0	4.9230120278595624E-5	7.0	7.173327417808807E-5
8.0	3.701396367109952E-5	7.5	6.957038222062828E-5
9.0	3.3140565570934525E-5	8.0	7.230105369338604E-5
10.0	2.956466587870164E-5		

**7500 G**		**1 T**	
T	tao	T	tao
2.1	9.586193142962328E-4	2.1	0.0019399758353298865
2.3	8.008100580817459E-4	2.3	0.0017483637385165237
2.5	6.884768253392804E-4	2.5	0.0016029911424150895
2.7	5.959060682065348E-4	2.7	0.0015048424840793744
2.9	5.356789629798157E-4	2.9	0.0014277578789448982
3.1	4.763143631180578E-4	3.1	0.0013457059308046879
3.3	4.3499800243046823E-4	3.3	0.0012835621121895704
3.5	3.6739716081109604E-4	3.6	0.001201549413650063
3.9	3.2640235396426724E-4	3.9	0.0011236234506016452
4.2	2.9539904345705887E-4	4.2	0.001049044146591724
4.5	2.6104600990936274E-4	4.5	0.0010076814944425586
5.0	2.2622491216297514E-4	5.0	9.049585576181426E-4
5.5	1.911600113270554E-4	5.5	8.16920069532408E-4
6.0	1.766761223116008E-4	6.0	7.506952115892297E-4
6.5	1.5671215750867354E-4	6.5	6.736382332981689E-4
7.0	1.4881088177958832E-4	7.0	6.207110898633434E-4
7.5	1.2745185761540442E-4	7.5	5.605173922777145E-4
8.0	1.1842722121842252E-4	8.0	5.151975281660415E-4

1.5 T		1 T	
T	tao	T	tao
2.1	0.004521266261599925	2.1	0.0024685633530519835
2.3	0.004471670202739805	2.3	0.0026801679781751995
2.5	0.004274428233876099	2.5	0.002342547426387692
2.7	0.004249923568881967	2.7	0.002263389193269567
2.9	0.004370860515314915	2.9	0.0025728917785061353
3.1	0.004081064307102769	3.1	0.0023195172357407946
3.3	0.004088383231500028	3.3	0.0033125625229337486
3.6	0.003775278062701743	3.6	0.002385576640208957
3.9	0.003510537469546186	3.9	0.002313248179091591
4.2	0.0032706324303379496	4.2	0.0022798798130038516
4.5	0.002996077568424316	4.5	0.0021227286730178974
5.0	0.0025806908618069752	5.0	0.0019008688972575497
5.5	0.0021890773681604698	5.5	0.0016714895733895582
6.0	0.0019122727669294843	6.0	0.0014562262992148165
6.5	0.0016426445424266478	6.5	0.00132365427919163
7.0	0.0014195209463692894	7.0	0.0011743402787031082
7.5	0.001239565972270273	7.5	9.861501046450779E-4
8.0	0.0010978568958907933		
2 T		3 T	
T	tao	T	tao
2.1	0.0024685633530519835	2.5	6.484877948310982E-4
2.3	0.0026801679781751995	2.7	6.428568865250932E-4
2.5	0.002342547426387692	2.9	6.61289292504669E-4
2.7	0.002263389193269567	3.1	6.733790112822956E-4
2.9	0.0025728917785061353	3.3	6.917345619013869E-4
3.1	0.0023195172357407946	3.6	6.609546880945694E-4
3.3	0.0033125625229337486	3.9	6.872264565370561E-4
3.6	0.002385576640208957	4.2	6.880617934521366E-4
3.9	0.002313248179091591	4.5	6.948683957244183E-4
4.2	0.0022798798130038516	5.0	6.526431003051732E-4
4.5	0.0021227286730178974	5.5	6.245212660850303E-4
5.0	0.0019008688972575497	6.0	6.036422873254267E-4
5.5	0.0016714895733895582	6.5	5.552853467796282E-4
6.0	0.0014562262992148165	7.0	5.26582405483164E-4
6.5	0.00132365427919163	7.5	4.918295360254838E-4
7.0	0.0011743402787031082		
7.5	9.861501046450779E-4		

Table S7. Best-fit parameters of the model used to reproduce the field dependence of the magnetization relaxation for **2** (Equation 2 in the main text).

T **(K)**	***C*** **($T^{-4}s^{-1}$)**	***d*** **(ms^{-1})**	***e*** **(T^{-2})**	***f*** **(T^{-2})**
**2.3**	10 (3)	560 (24)	0.05	1022 (89)
**2.5**	14(3)	671 (32)	0.01	951 (50)
**2.7**	18(5)	795 (34)	1 x 10-3	874 (82)
**2.9**	14(6)	926(40)	0.04	1139 (80)
**3.1**	13(7)	1081(60)	2.62 x 10-4	898 (68)
**3.3**	15(7)	1280 (53)	4.65 x 10-4	1143 (33)
**3.6**	15 (8)	1431 (79)	5 x 10-3	1008(86)
**3.9**	13 (7)	1823(63)	1 x 10-4	1176 (37)
**4.2**	12(11)	1965(104)	0.02	1181 (48)
**4.5**	10 (14)	2305(91)	1 x 10-4	1174 (58)
**5**	11 (11)	2926 (98)	1 x 10-4	1000 (36)
**5.5**	11(16)	3716 (131)	1 x 10-4	1116(38)
**6**	14(25)	4386 (201)	1 x 10-4	1506 (49)
**6.5**	12 (24)	4881(305)	1 x 10-4	1611 (42)
**7**	15(33)	5719 (442)	5 x 10-3	1643 (51)
**7.5**	15 (139)	6353 (522)	5 x 10-3	1964 (45)

C parameter as it is expected shows a strong uncertain for higher temperatures that becomes poorly significative above 4.5 K; the important *d* parameter increases the uncertain for higher temperatures but remain below 8% as maximum; *e* parameter shows a value practically zero for all temperatures.

References

(1) Mertes, K. B. Crystal and molecular structure of a silver (II) complex with a synthetic macrocyclic ligand. *Inorg. Chem*. 1978, 17, 49-52.

(2) SHAPE v.2.0. Lunell, M.; Casanova, D.; Cirera, J.; Alemany, P.; Alvarez, S. Barcelona **2010**. The program can be obtained by request to the authors.
